# Experimental Development of an Injection Molding Process Window

**DOI:** 10.3390/polym15153207

**Published:** 2023-07-28

**Authors:** Mason Myers, Rachmat Mulyana, Jose M. Castro, Ben Hoffman

**Affiliations:** 1 Industrial and Systems Engineering, College of Engineering, Columbus Campus, The Ohio State University, Columbus, OH 43210, USA; myers.1828@osu.edu (M.M.); mulyana.1@osu.edu (R.M.); 2 Honda of America Mfg Inc., Raymond, OH 43067, USA; ben_hoffman@na.honda.com

**Keywords:** injection molding, process windows, simulation, controllable process variables, performance measures

## Abstract

Injection molding is one of the most common and effective manufacturing processes used to produce plastic products and impacts industries around the world. However, injection molding is a complex process that requires careful consideration of several key control variables. These variables and how they are utilized greatly affect the resulting polymer parts of any molding operation. The bounds of the acceptable values of each Control Process Variable (CPV) must be analyzed and delimited to ensure manufacturing success and produce injected molded parts efficiently and effectively. One such method by which the key CPVs of an injection molding operation can be delimited is through the development of a process window. Once developed, operating CPVs at values inside the boundaries of the window or region will allow for the consistent production of parts that comply with the desired Performance Measures (PM), promoting a stable manufacturing process. This work proposes a novel approach to experimentally developing process windows and illustrates the methodology with a specific molding operation. A semicrystalline material was selected as it is more sensitive to process conditions than amorphous materials.

## 1. Introduction

Injection molding is a manufacturing process for plastic parts, that can be found in almost any industry, whether it be electronics, healthcare, consumer goods, or automotive. The global plastic injection molding market is expected to reach over 266 billion dollars by 2030 [1]. The goal of Injection Molding, like any other manufacturing process, is to produce parts or products efficiently and effectively. Several factors involved in this process can significantly affect the resulting products. One such way to increase the performance of the injection molding process is to construct an operating envelope in which key controllable factors, or variables, have been delimited. With the overall goal of producing acceptable values of the relevant performance measures of the specific injection molding process [2,3,4,5,6]. This operating envelope is commonly called a process window. This paper focuses on discussing a novel approach based on experiments through which an injection molding process window can be constructed.

The following steps were followed to develop the process window:

1. Identify and establish key Controllable Process Variables (CPV) for the process, including material attributes, machine settings, and mold/part conditions;
2. Identify the relevant performance measures for the specific process, such as part quality indicators and mechanical property values;
3. Develop a process window to delimit the controllable process variables such as packing time, injection speed, mold temperature, melt temperature, and packing pressure;
4. Supplement the experimental results via simulation to illustrate conditions for which the use of a less desirable location in the process window has to be selected. The software utilized in this study was Moldex3D (2021R2OR 64-bit).

These steps are the basis of the methodology developed, and each of them is discussed in detail in the following sections.

Each stage of the injection molding process critically influences the performance measures of the final product. It was desired to identify a region where the relevant controllable process variables within these stages have a range or envelope of operation in which acceptable parts are produced. This is the so-called process window. This research focused on developing a novel, industrially relevant, organized approach to define this acceptable range or process window. This window’s exact shape and size will depend not only on the polymer part to be molded but also on the machine and the quality of the mold used. The specific values of the process window are also material dependent; however, the approach presented here towards developing the process window should be applicable to other materials. We selected semicrystalline materials as they are more sensitive to process conditions than amorphous materials.

## 2. Materials and Methods

**Figure 1:**
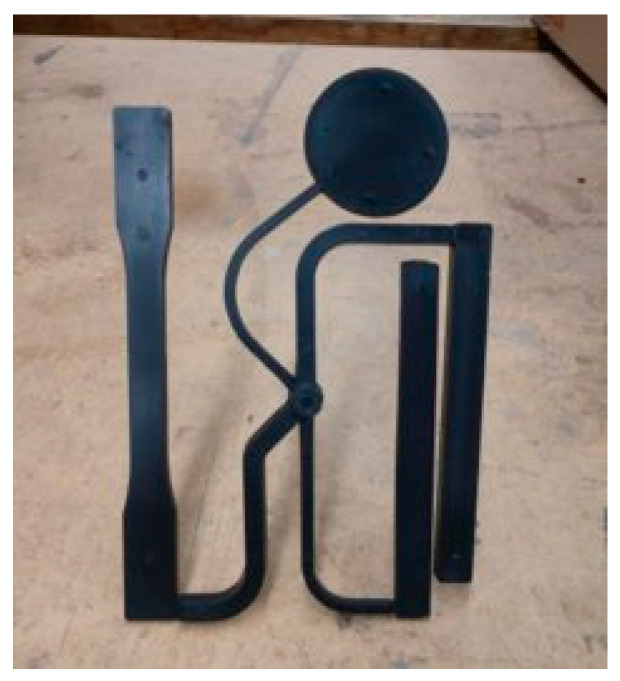
Picture of a molded ASTM polymer part.

The machine used throughout this research was a Sumitomo 180-ton Injection molding Machine (SG180M-HP, Tokyo, Japan). The cavity of the mold used in this study produced the parts utilized for measuring mechanical properties as indicated by the American Society for Testing Materials (ASTM). The mold material was tool steel, with several cooling lines running throughout both halves of the mold. When ejected from the mold, a sprue and cold runner system connect the 4 test samples utilized for mechanical testing, as shown in Figure 1.

The 4 test samples produced include a tensile bar, two flexural bars with differing thicknesses of 3 mm and 6 mm, and a disk with a diameter of 50 mm. These samples can be used to conduct a variety of tests, including tensile testing, 3-point bending, and impact testing such as the Izod or Dupont tests. The material used in this work was polypropylene, produced by Advanced Composites. The application of this material in industry was for automotive body panels.

The mechanical properties were measured with an INSTRON 5569 Dual Column Table Top Load Frame. The INSTRON has a maximum speed of 500 mm/min and the minimum speed that can be used is 0.005 mm/min. The load capacity is 50 kN [7]. A speed of 50 mm/min was used, which is larger than the one recommended by the ASTM but is the speed used by automotive manufacturers. A TA Q20 Differential Scanning Calorimeter (DSC) was used to measure the crystallinity of the modified polypropylene samples [8,9,10].

When operating an injection molding machine, several settings must be selected to produce parts successfully. The definition of a “successful” part depends on the desired performance measures for the specific molding operation and will be outlined later, before the development of the final process windows. The settings that will be discussed here are those that are most important to the overall process and are used as the controllable variables of the process window to be developed. These key variables are melt temperature, mold temperature, injection screw speed, packing pressure, packing/cooling time, clamping force, and shot size. These variables are important in any injection molding operation. This research found it convenient and beneficial to separate these variables into tiers to help streamline and focus the development of the process window. Table 1 presents this concept.

The Primary Control Variables of mold temperature, melt temperature, and packing pressure were deemed most important for this molding operation as they were the variables used to construct the boundaries of the process window.

The control variables of melt and mold temperature and their effects on the resulting part and operation closely interact with each other. The melt temperature must be high enough to allow the material to fill the mold completely. On the other hand, the mold temperature can be adjusted to benefit cycle time. Most likely, both variables affect the properties of the final part. Simply put, a balance must be achieved to produce parts effectively and efficiently. The last primary variable is packing pressure. This pressure ensures the mold cavity is fully filled and reduces thermal shrinkage as the material cools in the mold. It is important to note that the packing pressure values reported in this work are not the pressure on the polymer inside the mold cavity but the pressure setting entered into the machine, which corresponds to the hydraulic pressure. We measured the corresponding pressure inside the cavity to relate the “real values” to the machine settings. For example, a machine packing pressure of 2.07 MPa corresponds to a cavity pressure of about 41.37 MPa [11]. The approximate multiplier to calculate part pressure from machine pressure settings is twenty [11].

Due to the effects of these 3 primary variables on the overall injection molding process and part quality, understanding their relationship is critical to delimiting a process window to produce acceptable, defect-free parts. For the secondary control variables, that is, injection screw speed and packing/cooling time, the values chosen can have serious implications on the resulting polymer part and were analyzed before the primary control variables were studied. However, after these variables were delimited, specific settings or values were selected and kept constant throughout the process window’s development. Lastly, the tertiary control variables are those that can affect a particular molding operation but are easier to establish. The clamping force must be large enough to hold the two mold halves together but not too high to damage them. The shot size was determined by the volume of the molded part. These tertiary control variables required little analysis and were held at the same settings for the entirety of the process window’s development.

**Figure 2:**
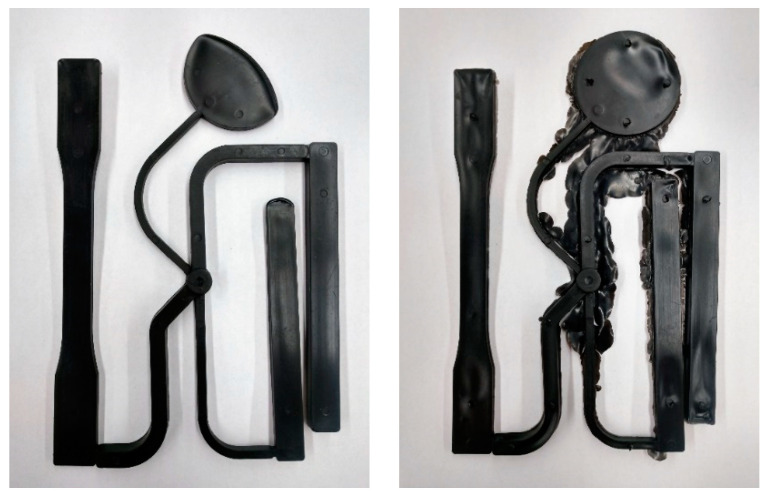
Examples of defect types: short shot, flash, and sink marks.

As the focus of this research was to develop a method to establish a process window to produce thermoplastic injection molded parts, it is necessary to understand the concept of such a window. A process window is a region that delimits the controllable process variables so that inside the boundaries of this region or window-like shape, acceptable parts, that is, parts that comply with the desired performance measures, can be molded. Operating outside of the process window will produce molded parts that are unacceptable due to defects such as flash, sink marks, and short shots. Examples of such defects are shown in Figure 2 [7].

**Figure 3:**
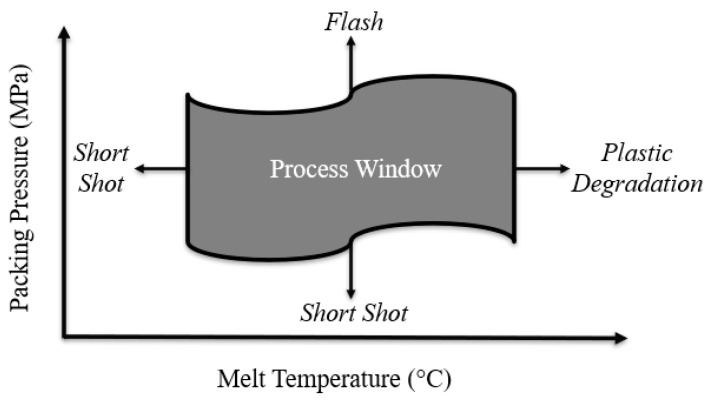
Example of a process window considering melt temperature and packing pressure.

Figure 3 visually illustrates a process window that considers the controllable process variables of melt temperature and packing pressure. If the melt temperature is too low, the plastic will solidify before the mold is filled, producing a short shot. On the other hand, if the temperature is too high, plastic degradation may occur. If the packing pressure is too low, a short shot could occur, and if the packing pressure is too high, leakage (flash) will occur.

When the selected values of the controllable process variables are near the center of the defined window, the part quality will be less influenced by undesired variations in the process variables. Variations due to unforeseen conditions likely caused by the molding environment. Therefore, the use of a process window in injection molding promotes a stable and more predictable manufacturing process [12]. Although a particular process window will vary from one production setting to another, the method by which these windows can be found has the same steps and considerations. A method that can be used for constructing such a window will be presented throughout.

## 3. Experimental

The first step in developing an injection molding process window was to identify, define, and determine the base values of the controllable process variables. The manufacturing of polymeric parts via injection molding is a complex problem. Each Controllable Process Variable (CPV) can have an impact on the resulting polymer part. Because of this, it is best to simplify and determine which variables will be changed and which will remain constant based on the goals of the process window being developed. This is the reason we divided them into primary, secondary, and tertiary tiers. It is best to only consider settings critical to the specific molding operation, as too many variables will greatly complicate the development method. We will discuss what CPVs were identified, how their values were found, and if they were kept as constants or varied as part of the process window.

Several values or ranges of the CPV were defined based on known information about the specific molding material, mold, and injection molding machine, as these factors will not change throughout the development process. Table 2 provides the reference for each of these variables and lists their selected ranges or values.

Other variables can also be delimited, but it took more analysis to properly determine their values with respect to the specific operation. These variables are listed in Table 3 and will be defined and discussed in more detail.

The CPVs of mold closed time, injection screw speed, preliminary packing pressure, and packing/cooling time required further analysis to determine their respective values. These variables will likely have the most variation from one injection molding operation to another. Therefore, to determine the best settings for these variables, a specific analysis was conducted to isolate the influence of each variable and determine its value regarding the unique molding operation. The values found by this analysis will be different from one operation to another; however, the methods by which they were found will be similar. Although most of these values were later treated as constants in the development of the process window, to find their best values, they were each treated as independent variables during their specific analysis step. A discussion on how each of these variables was defined and the method used to determine their values is presented.

Table 4 presents the various settings that each of the CPVs were set to during this analysis stage [7].

The first analysis conducted was to delimit the value needed for mold closure time, that is the time needed for the part to become solid enough so that it can be demolded without any blemishes. The approach we recommend and that is used in our group [2,7] is to start by calculating the conduction time ($t_{CT}$), that is:

(1) $$t_{CT}\;=\frac{h^{2}}{\alpha }$$

where: $h\;=\frac{partthickness}{2},\alpha =ThermalDiffusity$.

Part thickness is the largest thickness of the polymer part to be molded. This time is a naturally occurring time when making the heat conduction equation dimensionless and represents the time needed for the temperature in the center of the part to be such that its value minus the mold temperature becomes 10% of the maximum temperature difference (melt temperature minus mold temperature) if one assumes one-directional heat transfer and constant mold wall temperature. We suggest and have used it both in our lab and in our interactions with industry as an initial value, in general, a conservative number that can be decreased using experiments. For this case, we kept the calculated value as the mold closed time. This value will later be subdivided between packing time and cooling time. Once the packing pressure does not affect the part quality, the part is kept in the mold without packing pressure. That way, the screw can start retracting for the next cycle, and the mold stresses are minimized.

**Figure 4:**
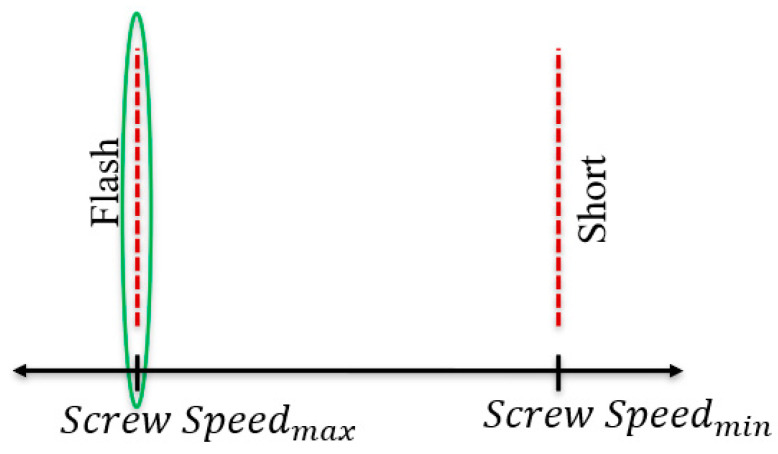
Injection screw speed spectrum with defect boundaries.

The injection screw speed of an injection molding machine is the speed at which the screw or plunger moves during the injection stage. Correctly determining the value of this setting is important, as injecting too fast will cause flash, but injecting too slowly will cause the melt front to solidify before the part is completely filled, also known as a short shot. The goal of this analysis was to find a maximum injection speed before flashing occurs to reduce the total cycle time but also produce an acceptable part. This concept is represented and discussed below (Figure 4).

Again, the goal of these experiments was to find the injection screw speed just on the boundary before flashing occurs. With regards to this research, it was unnecessary to explore the right-hand side of the spectrum, as injecting the material slowly into the mold has no real benefit and would slow cycle times. However, slower injection times may, in some cases, produce better results, such as surface finish, and therefore should be explored if applicable [13].

Several runs were conducted to establish the proper setting for injection screw speed. To focus on the injection of the material and the screw speed, values associated with packing were minimized by selecting a very low packing pressure of 10 psi (0.07 MPa) and allocating zero seconds of the calculated conduction time to packing (the entire 52 s was set to cooling). Our group has found that using a cushion position that is ten percent of the total shot size (0.210 inches (53.34 mm)) is good practice. Using the settings above, molded parts were produced and visually analyzed. Screw speeds ranging from 0.2 in/s to 10 in/s (5.1–254 mm/s) were tested as shown in Table 4.

After the completion of these trials, a visual inspection [7] of the parts was conducted to determine the best value for this setting. It was found that at a speed less than or equal to 2.0 in/s (50.8 mm/s), the mold would be filled without flash. The setting of injection screw speed was thus established at this constant value during the process window development trials and considered a secondary control variable.

Although packing pressure will be used as a primary CPV during the development of the process window itself, it was necessary to find a “preliminary” setting for this CPV to select values for the other settings. The procedure to delimit this initial packing pressure was to run injection molding trials and vary the packing pressure. By doing so, a preliminary process window related to packing pressure was found in which acceptable maximum ($P_{max}$) and minimum ($P_{min}$) packing pressures were established. Pressure outside this window would either cause the polymer part to flash or produce a short shot [7]. From this process window, an average of $P_{min}$ and $P_{max}$ was calculated. This $P_{avg}$ was the value used as the preliminary packing pressure in the remaining analysis trials. The experiments and analysis conducted to preliminarily delimit the packing pressure are as follows:

To delimit and isolate the packing pressure, several of the other molding variables were set to specific values. For example, the melt temperature was set to the middle value of the range provided by the material supplier. With regard to time, the overall mold closing time was kept equal to the calculated conduction time of 52 s. At this stage, this entire length of time was allocated to packing time ($t_{pack}$), and the cooling time ($t_{cool}$) was set to zero, again shown in Table 4. During this analysis, samples were produced with packing pressures ranging from 100 psi to 750 psi (0.69–5.17 MPa). Each polymer part produced was then analyzed to find defects and determine the $P_{max}$ and $P_{min}$ of the preliminary packing pressure window. To find this range, a combination of visual inspection and measurements was utilized.

**Figure 5:**
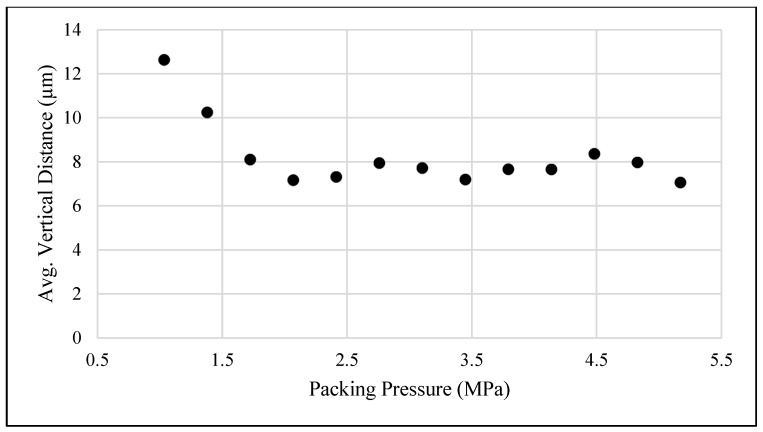
Average vertical distance versus packing pressure measured by a profilometer.

To determine the minimum boundary, both visual inspection for defects and the measurement of the surfacing profile of the molded parts using a profilometer were utilized. The results of these measurements are shown in Figure 5.

As can be seen from Figure 5, as packing pressure increased, the vertical distance from the highest outside edge of the measured piece to the lowest middle point became stable. From these measurements, it can be seen that 1.7 MPa is the initial packing pressure value where the surface profile began to level out, indicating the part was completely filled. Additionally, visually inspecting the molded parts found that all corners and end pieces were completely filled, and no sink marks were seen, beginning at this value. Therefore, a minimum packing pressure of 1.7 MPa was determined.

**Figure 6:**
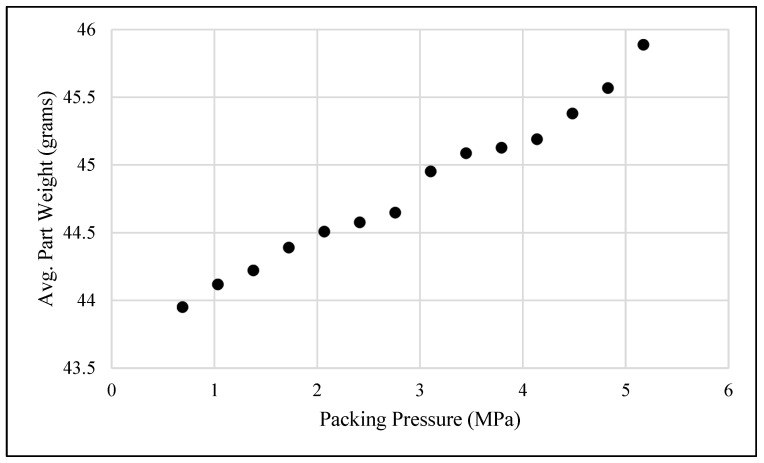
Average molded part weight versus packing pressure.

To determine the maximum limit for the preliminary packing pressure, several other measurements were utilized. One such measurement was the total part weight. These results are shown in Figure 6.

Looking at Figure 6, which displays the total weights of the mold parts in grams, it can be seen that from about 2.76 to 3.79 MPa, there was an increasing trend in the weights of the molded parts. This was taken as an indication that the flash being seen was becoming more significant and therefore unacceptable, as indicated in Figure 2. It was also observed that as the packing pressure increased, the final cushion position of the injection screw significantly changed. At around 2.76 MPa, a large drop-off from the machine setting of 0.210 inches (5.33 mm) was seen. This indicated that the screw was going well past its desired final position and pushing extra material into the mold, causing significant flash when higher packing pressures were introduced. Combining these results with visual inspection, a maximum value of 3.1 MPa was determined.

**Figure 7:**
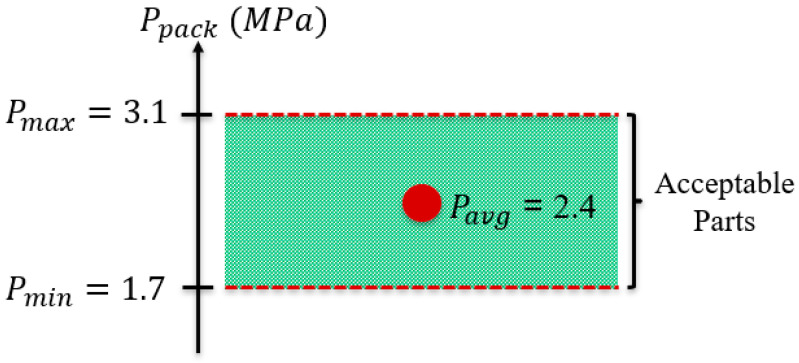
Determined preliminary packing pressure process window.

From the above analysis, the resulting process window shown in Figure 7 was developed.

From these findings, an average packing pressure of 2.4 MPa was calculated. This $P_{avg}$ was used in the additional trials to determine the values of the remaining non-primary controllable variables. Once these values were established, packing pressure was treated as a primary controllable process variable during the development of the final process window.

The calculated conduction time of 52 s is subdivided into packing time and cooling time. Determining the ratio between these two periods was important because, after the point when the packing pressure has no effect, it is desirable to switch from packing to just cooling so the screw can start retracting, as well as to avoid unnecessary stresses on the mold. The process undertaken to delimit this ratio, its justification, and the resulting times are discussed below.

First, a range of ratios between $t_{pack}$ and $t_{cool}$ was selected with respect to the total conduction time. At each of these ratios and the settings shown in Table 4, injection molded samples were collected, inspected, and measured.

Utilizing both the measurement of part weight and surface profile via profilometer [7], it was found that the packing-to-cooling time ratio must be greater than 20 to 80, or 10.4 s and 41.6 s, respectively. To support the results from part weight and shrinkage measurements [7], tensile testing of the parts was conducted. For each of the six-time ratios, tensile testing was completed. These results are presented in Table 5.

With a combination of part weights, surface profiles, and tensile tests, the ratios were delimited. As stated, the results of the weights and profilometer indicated that the use of ratios less than 20:80 was not desirable [7], and this is supported by the tensile test results. The ratio of one hundred percent packing time and zero percent cooling is also not acceptable, as it did not allow the screw to start retracting early enough to prepare material for the next cycle, thus slowing production. Additionally, by completing a conduction time analysis on the part’s cylindrical sprue, it was found that packing for more than approximately 45 s would not be beneficial as a large percent of the sprue cross-section, or entry into the mold, will have already solidified.

Combining all the experimental evidence, it was determined that the best packing time to cooling time ratio was 40 to 60 percent, respectively, as shown in Table 6. This conclusion was reached based on several factors. Firstly, the samples produced under this ratio visually appeared to be the best. The weight and profilometer measurements also supported this ratio, as the samples were shown to be completely filled. Additionally, this ratio produced the best mechanical properties, with the highest tensile stress at yield and break, along with increased ductility (tensile strain at break).

These secondary settings would remain constant throughout the development of the process window discussed in the remaining sections.

## 4. Results and Discussion

With the secondary and tertiary process variables established, we proceeded to develop the process window. As previously stated, mold temperature, melt temperature, and packing pressure were the primary CPVs that defined the boundaries of the process window. Table 7 summarizes all the controllable variables and their values as found previously.

As the primary CPV defined the boundaries of the process window, their effect on the performance measures was evaluated next. A full factorial for both temperatures at three levels was conducted. Ten samples at each setting were collected. The values of the melt temperature used are 179.4, 193.3, and 210 °C. The values for the mold temperatures are 26.7, 37.8, and 48.9 °C. Packing pressure was varied at each temperature combination until a minimum and maximum value were found for each. Nine unique temperature combinations were tested, all with various levels of packing pressure. To thoroughly construct the process window, a total of more than 750 parts were modeled and analyzed. The first window that was developed was based on visual inspection.

**Figure 8:**
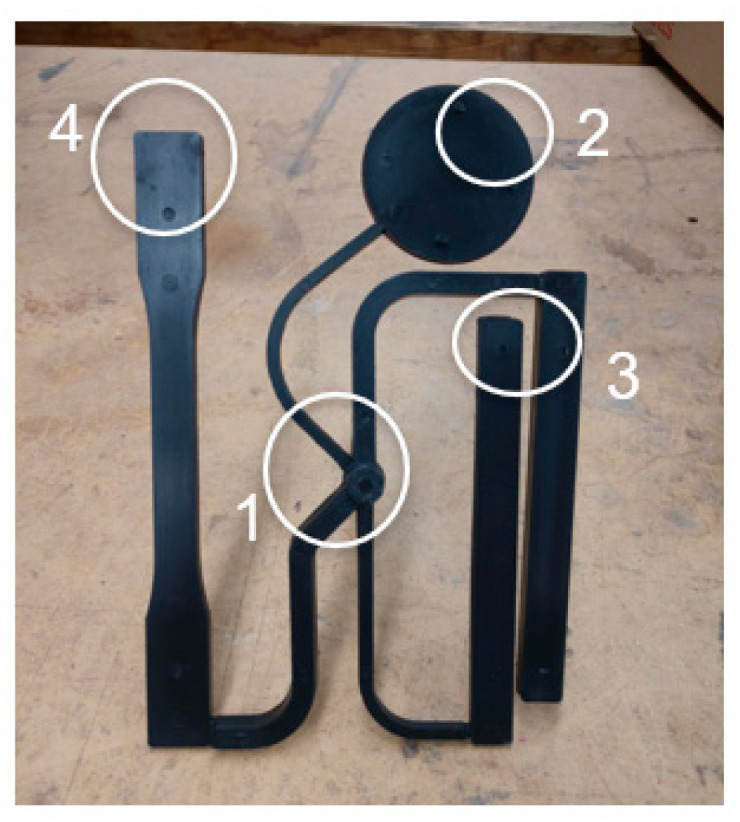
Visual quality standard zones (labeled 1–4) on molded parts.

Quality standards [7] were established in order to define what constitutes an acceptable part. These standards will likely vary on a case-by-case basis, but they must be defined to keep the process repeatable and measurable. In the case of this research, visual quality standards were developed to focus on key areas or zones of the part being produced. Figure 8 highlights these areas.

Within each of the respective locations of the polymer part, a visual inspection for defects was conducted, looking for such things as flash and sink marks. As depicted in Figure 8, zones 2, 3, and 4 were inspected for complete fill and a lack of shrinkage in corners and edges. Additionally, zones 1 and 3 were inspected for significant flashing. With these quality standards defined, they were used to analyze parts produced at various packing pressures and temperatures. If the quality standards defined were not met by particular molded samples, then the associated molding condition was deemed unacceptable. From this analysis, the boundaries, or minimum and maximum acceptable packing pressure, for each of the nine temperature combinations were found, therefore producing a visual process window.

**Figure 9:**
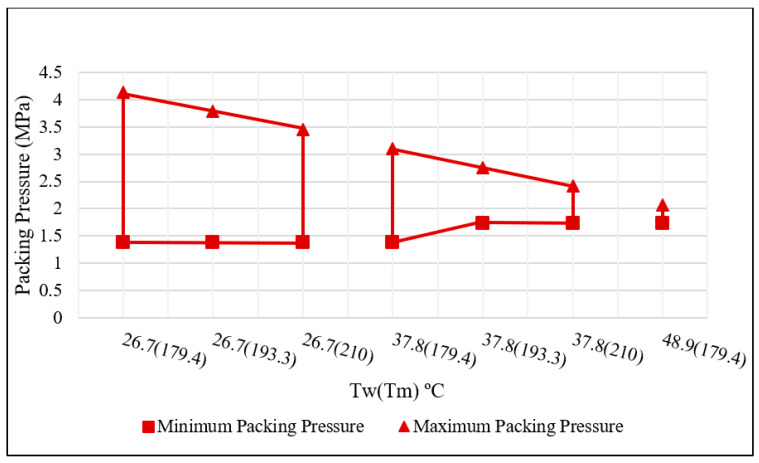
Visual process window.

The resulting visual process window was developed by utilizing the defined quality standards and inspecting the molded parts produced [7]. Based on the standards, a minimum and maximum packing pressure value for each temperature combination—mold temperature and melt temperature—were found. The visual process window was produced by overlaying these ranges onto a single plot. Figure 9 represents the visual process window of this particular molding process.

Several features of this resulting process window must be discussed, along with a justification of the resulting window based on fundamental principles. Firstly, it may be noted that only seven temperature combinations were included within the developed window when nine such combinations were molded. This occurred because no parts produced above the mold and melt temperature settings of 48.9 °F and 179.4 °C were found to be acceptable based on the quality standards. Because these molded parts were found to be visually defective, they were also filtered out or eliminated from the later mechanical property analysis.

In terms of fundamentals, the resulting visual processing window was justified. The steady decrease in acceptable maximum packing pressure (the upper bounds of the window) was supported by the fact that as the temperature increased, the thermoplastic material became less viscous and therefore increased potential leakage, causing the defect of flashing. This was observed on molded parts above the maximum boundary. The acceptable minimum packing pressure was affected by the increasing temperature as well. The increased temperature also caused increased shrinkage as the part cooled in the mold. This explains the increase in the minimum packing pressure values once mold temperatures were at or above 37.8 °C. Parts below the designated minimum were observed to have sink marks or were short in filling.

From the trend seen in the process window, it can be noted that mold temperature appears to have a greater effect on the developed window and part acceptability. The packing pressure range for each temperature combination was greatly reduced as mold temperatures increased. This relationship is most likely material and part dependent. 

The left side, the side with a lower mold temperature, is more robust; that is, it would allow for more uncontrollable variations in your molding environment and still enable the production of acceptable parts. Additionally, because the mold temperature is low, the molded part cools faster and can be ejected sooner, promoting a shorter cycle time. For this particular molding operation, the right side of the visual process window is less desirable and may only be used as a boundary of limitation. However, depending on the complexity of the part, the right side or higher mold and melt temperatures may be necessary, as illustrated with an example later.

The process window, based on visual inspection, is a great tool to delimit the relevant CPV, and select the most robust region of the CPV domain. However, it was believed that the visual inspection used to construct the injection molding process window was only part of the whole development process, and more analysis was needed to promote and produce a more robust solution. This is particularly true for semicrystalline materials and less important for amorphous materials [8].

Since our material is semicrystalline, to develop a more robust process window, analysis of the mechanical properties of the parts produced within the visual process window was necessary. Although the visual process window developed indicates to a molder how to operate their machine in order to produce visually acceptable parts, it does not include the effect on the mechanical properties of the polymer part. The approach in industry is to define acceptable parts based on visual inspection; mechanical properties are rarely considered for specific parts after they are molded [14]. This could lead to unacceptable parts, in particular for semicrystalline materials [8]. This research aimed to further the analysis beyond just the visual process window and develop a more refined process window that took mechanical properties into consideration. Mechanical properties, in particular ductility, may be affected by process conditions for semicrystalline materials [8].

The desired mechanical properties of a particular injection molded part will vary from one product to another. In the case of this analysis, experiments were conducted to determine the tensile properties of the samples produced. With the visual process window already defined, the molding conditions and the number of samples for the tensile test were decreased, as polymer parts deemed visually unacceptable were not tested mechanically. The two properties that will be discussed to generate a more refined process window are tensile strain at yield and tensile strain at break, or ductility. The results of these two properties are presented and discussed below (Table 8 and Table 9).

Looking at the tensile strain at yield results, the effects the CPV has on this property become clear, as higher values indicate that the parts deformed to a greater degree before yielding or the start of plastic/permanent deformation. In terms of packing pressure, the samples produced with increasing pressure result in higher tensile strain in all combinations of mold and melt temperatures. Additionally, with regards to temperature, lower melt temperatures produce samples with increased tensile strain. In all three cases of different mold temperatures, the lower range of the melt temperature (179.4 °C) produces the highest strain values. Lastly, it was observed that overall, the mold temperature of 26.7 °C produced higher tensile strain results. In conclusion, these results indicated that the mechanical property of tensile strain at yield benefited from higher packing pressure and lower mold and melt temperatures, as it takes longer or more deformation to cause parts molded at these conditions to yield.

Table 9 presents the results of the tensile strain at break or the ductility of the various samples that were tested mechanically. These results provide similar conclusions to those found for the tensile strain at yield. Higher ductility is achieved by samples that were both molded at higher packing pressures and at lower mold and melt temperature combinations. With high ductility, parts molded under these conditions will deform but not break or fail easily.

The analysis of tensile strain at yield and at break indicates that the percent crystallinity of the samples at lower temperatures is most likely lower, and thus they are more ductile. This has been corroborated by Differential Scanning Calorimetry (DSC) [8,9,10]. These results justify the conclusion that further analysis of the mechanical proprieties benefits the creation of a more refined injection molding process window. The findings discussed indicated that improvements could be made to the visual process window originally found to produce a window that promotes both visual and mechanical success. This may not be the case for amorphous materials [8,9].

Recall that it was found that using lower mold and melt temperatures produced better tensile strain properties. Combining both the findings of the visual inspection process window and the tensile strain testing (mechanical properties), a final refined process window was produced.

**Figure 10:**
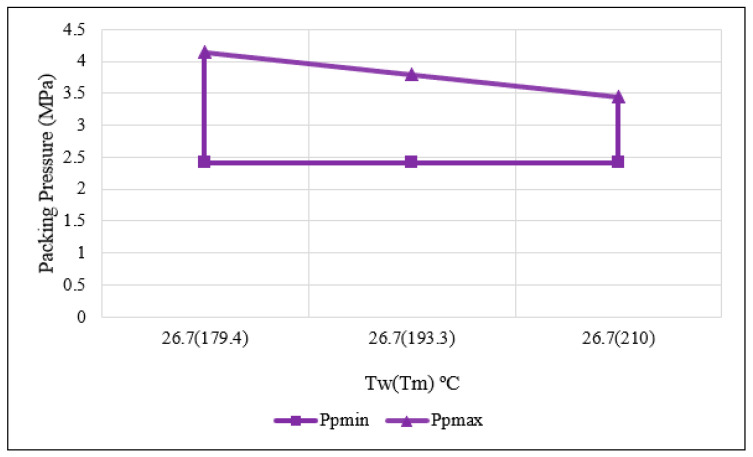
Final refined process window.

This process window shown in Figure 10 suggests operating at a mold temperature of 26.7 °C and a melt temperature of 179.4 °C to 210 °C. Additionally, packing pressure should be set between 2.41 and 4.14 MPa, depending on the temperature. This process window will promote a more stable and predictable injection molding operation by reducing the impact of undesirable variation in the molding environment and also improving the resulting parts’ visual and mechanical properties.

**Figure 11:**
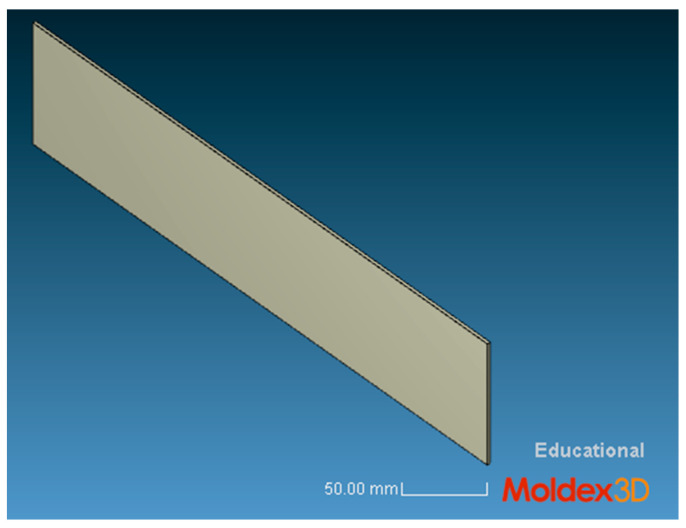
A thin plate model (0.375 m × 0.1 m × 0.002 m) visualized with software Moldex3D.

For complicated parts, in particular parts with thin sections, it may be necessary to mold at higher temperatures in order to completely fill the part or to use the least desirable region of the process window. We illustrated this with a simple example, where we analyzed the filling of a flat plate with increasing length using a fan gate, as shown schematically in Figure 11. The Computer Aided Engineering (CAE) software used in this case is Moldex3D.

The specifics of this example were to construct a flat plate of a certain length. Then we ran a filling stage analysis using Moldex3D to determine if the temperature allowed the flat plate to successfully fill. If the plaque was successfully filled, a new plaque of longer length was then analyzed. If the temperatures used did not allow the mold to fill completely, then the temperature values were increased. This process was repeated for a variety of flat plate lengths and used the mold and melt temperature ranges recommended by the material supplier and used in the original visual processing window.

With the case study defined, several simulations were completed. The results of these runs are shown in Table 10.

As can be seen from the table, plates of shorter lengths filled successfully at lower mold and melt temperatures. However, as the plate length increased, higher temperatures, specifically melt temperatures, were required to fill the mold completely.

**Figure 12:**
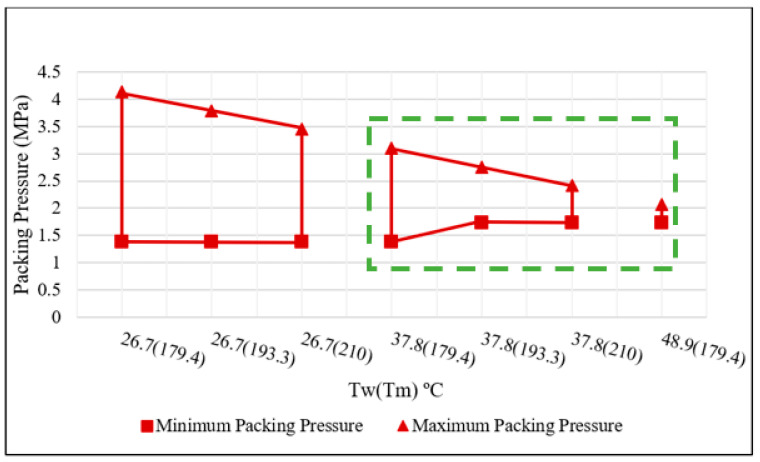
Visual process window is highlighted with simulation results.

The results indicated that the temperature ranges found to be the best for promoting visual and mechanical success during the experimental development of the process windows did not allow for complete filling in certain cases of the thin plate molds. Although it was found earlier that visual inspection and mechanical properties benefited from lower melt and mold temperatures, the simulations show that such temperatures may need to be increased in order to fill the mold when considering, large thin-walled parts. The right side, or the side representing higher temperatures of the visual process window shown in Figure 12, was better justified by this case study. We selected a flat plate for simplicity and easier discussion; however, we could have also performed the same analysis with the ASTM mold. Whereas before the right side of the process window was only used as a boundary of limitation, the simulations conducted indicate that although this area of the process window is not as robust, it may be necessary in order to produce fully filled parts. Again, it must be stated that the shape and size of a particular process window and the values of the key CPV will vary from one operation to another.

Molding thermoplastic parts that are both visually appealing and mechanically sound is desirable. However, the specific material and part dimensions of a particular operation may limit these quality factors. The simulations conducted as a part of this case study indicated that in order to successfully fill a thin plate, areas of the developed process window with higher mold and melt temperatures may have to be utilized, as indicated in Figure 12.

## 5. Conclusions

This research presented a method for the experimental development of an injection molding process window. By following each stage of the injection molding process, key controllable process variables were able to be isolated and analyzed. Using this method and defined quality standards, a visual process window was first found. This window focused on obtaining parts with acceptable appearance. In addition, this work proposed that this visual process window was only part of the solution, for semicrystalline materials in particular, and that there was a need to include mechanical testing as part of developing the process window. Through tensile testing, a more refined process window was developed. This refined window allowed for parts with both an acceptable appearance and adequate mechanical properties to be produced [8].

A special case study utilizing simulation was also presented, which helped to justify the use of specific regions of the experimental process windows, even if they were less robust from the visual and mechanical properties point of view.

The proposed approach can be summarized as follows. First, identify and establish key controllable process variables, including material attributes, machine settings, and mold/part conditions. Once this initial information is collected, determine the key performance measures and develop quality/measurement standards for the particular operation. Next, using part and material characteristics, calculate your conduction time ($t_{CT}$). Then, by following the order of each of the stages in the injection molding process, isolate and analyze each CPV to determine its range or value. Vary relevant controllable process variables such as mold temperature, melt temperature, and packing pressure to produce a large sample of molded parts. Using the produced molded parts and the defined performance measures, develop a visual process window. Lastly, refine the process via mechanical property analysis.

The seven steps above represent the general method used to experimentally construct the process windows presented and discussed in this work. It is suggested that the use of these steps will allow for the development of process windows in other injection molding operations. Again, it is important to note that the specific values presented during this work correspond to the particular molding process investigated (mold/part, machine, and material). Such values will likely differ from one molding process to another. This work is intended to present and provide a more standardized and thorough procedure for experimentally developing injection molding process windows. The steps highlighted above summarize the detailed research completed, and the results presented throughout showcase the success of the method developed.

## 6. Future Work

Firstly, it is suggested that the experimental method for developing injection molding process windows be tested further. Research using other materials or molding operations will help test the robustness of the method presented. This research would also help improve process window development strategies.

Due to the time commitment of conducting experimental research, it is also recommended that further analysis of simulations be pursued. In the case of this research, simulation is used as a supplementary tool to help better understand the experimental results. However, simulation software has promising potential to help develop injection molding process windows in a more direct way. In the future, with the help of the experimental results presented, strides in conducting more detailed simulation analysis to develop injection molding process windows will be possible. Utilizing the steps developed to construct an injection molding process window in this work as a guide, simulation can become the main tool used in order to avoid the limitations of physical trials. Process window development via simulation will allow us to test real parts where it is not possible to do experimental trials, such as an automotive production line. Work is currently being conducted in this area.

## Figures and Tables

**Table 1:** Control Variable Tiers.

**Primary Control Variable**	$\mathrm{Mold}\;\mathrm{Temperature}\;(T_{w}$)
	$\mathrm{Melt}\;\mathrm{Temperature}\;(T_{m}$)
	$\mathrm{Packing}\;\mathrm{Pressure}\;(P_{pack}$)
**Secondary Control Variable**	Injection Screw Speed
	$\mathrm{Packing}\;\mathrm{Time}\;(t_{pack}$)
	$\mathrm{Cooling}\;\mathrm{Time}\;(t_{cool}$)
**Tertiary Control Variable**	Shot Size
	Clamping Force

**Table 2:** Known Variables and Their Origin.

Variable	Origin	Value/Setting
$\mathrm{Mold}\;\mathrm{Temperature}\;(T_{w}$)	Material Supplier	80–120 °F (26.7–48.9 °C)
$\mathrm{Melt}\;\mathrm{Temperature}\;(T_{m}$)	Material Supplier/Molder Experience	355–410 °F (179.4–210 °C)
Shot Size	Part Volume	2.10 in (53.34 mm)
Clamping Force	Molder Experience	120 Ton (1067.6 kN)

**Table 3:** Variables Needing Further Analysis.

Variable
Mold Closed Time
Injection Screw Speed
$\mathrm{Preliminary}\;\mathrm{Packing}\;\mathrm{Pressure}\;(P_{pack}$)
$\mathrm{Packing}\;(t_{pack}$$)\;\mathrm{and}\;\mathrm{Cooling}\;(t_{cool}$) Time

**Table 4:** CPV Settings for Injection Screw Speed Trials.

CPV	Value
$\mathrm{Mold}\;\mathrm{Temperature}\;(T_{w}$)	80 °F (26.7 °C)
$\mathrm{Melt}\;\mathrm{Temperature}\;(T_{m}$)	380 °F (193.3 °C)
$\mathrm{Packing}\;\mathrm{Pressure}\;(P_{pack}$)	10–750 psi (0.07–5.2 MPa)
Injection Screw Speed	0.2–10 in/s (5.1–254 mm/s)
$\mathrm{Packing}\;\mathrm{Time}\;(t_{pack}$)	0–52 s
$\mathrm{Cooling}\;\mathrm{Time}\;(t_{cool}$)	0–52 s
Shot Size	2.10 in (53.34 mm)
Clamping Force	120 Ton (1067.6 kN)

**Table 5:** Tensile Test Mechanical Property Results (Average of 5 Samples Each).

$t_{pack}$$/t_{cool}$ (%)	Tensile Stress at Maximum Load [MPa]	Tensile Strain (Displacement) at Yield (Zero Slope) [%]	Tensile Stress at Yield (Offset 0.2%) [MPa]	Tensile Stress at Break (Standard) [MPa]	Tensile Strain (Displacement) at Break (Standard) [%]	Modulus Young [MPa]
100/0	19.279	12.122	8.843	14.271	145.491	909.661
80/20	19.353	11.950	8.510	14.864	254.9	992.231
60/40	19.367	11.447	8.443	13.596	118.440	1013.861
40/60	19.311	12.040	8.891	15.230	299.206	914.458
20/80	19.515	10.776	8.397	13.621	128.562	1019.808
0/100	18.169	7.246	8.831	15.54	38.87	941.908

**Table 6:** Established Packing and Cooling Time Ratio.

$t_{pack}$ (%)	$t_{cool}$ (%)	$t_{pack}$ (%)	$t_{cool}$ (%)
40	60	20.8	31.2

**Table 7:** Key Molding Variables and Determined Values.

Control Variable Tiers	Variable	Setting
Primary Control Variable	Mold Temperature	Controllable Process Variable (CPV)
	Melt Temperature	CPV
	Packing Pressure	CPV
Secondary Control Variable	Injection Screw Speed	2.0 in/s (50.8 mm/s)
	Conduction Time	52 s
	Packing Time	20.8 s
	Cooling Time	31.2 s
Tertiary Control Variable	Shot Size	2.10 in (53.34 mm)
	Clamp Force	120 Ton (1067.6 kN)

**Table 8:** Tensile Strain (Displacement) at Yield (%).

	$T_{w}({}^{\circ}C)$		26.7			37.8		48.9		
	$T_{m}\;({}^{\circ}C)$	179.4	193.3	210	179.4	193.3	210	179.4	193.3	210
**Packing Pressure** **(MPa)**	**1.38**	12.691	11.897	11.897	12.000	11.420	10.722	12.125	11.679	
	**1.72**	13.323	11.917	11.917	12.444	11.934	11.234	12.680	11.726	
	**2.07**	13.365	12.183	12.183	13.012	12.719	11.578	13.122		
	**2.41**	14.201	12.425	12.425	13.838	12.971	11.716			
	**2.76**	14.944	12.848	12.848	14.223	12.948	12.225			
	**3.10**	15.466	13.234	13.234	14.758	13.388				
	**3.45**	15.018	13.324	13.324	15.005					
	**3.79**	15.849	13.005	13.005						
	**4.14**	16.617	13.284							

**Table 9:** Tensile strain (Displacement) at Break (%).

	$T_{w}\;({}^{\circ}C)$		26.7			37.8		48.9		
	$T_{m}\;({}^{\circ}C)$	179.4	193.3	210	179.4	193.3	210	179.4	193.3	210
**Packing Pressure** **(MPa)**	**1.38**	118.557	180.703	165.851	150.313	148.742	188.623	146.582	175.032	
	**1.72**	134.699	182.579	141.534	177.650	155.772	151.928	160.629	158.783	
	**2.07**	136.120	181.355	151.462	140.261	155.257	149.869	135.287		
	**2.41**	125.384	247.614	262.226	145.024	187.168	145.354			
	**2.76**	299.574	371.187	271.889	158.577	234.021	210.268			
	**3.10**	320.251	340.745	370.961	198.609	316.248				
	**3.45**	344.143	373.189	371.243	237.441					
	**3.79**	371.349	336.061	400.026						
	**4.14**	325.522	377.526							

**Table 10:** Simulated Variables and Run Results.

Plate Length (m)	$T_{w}$ (°C)	$T_{m}$ (°C)	$T_{f}$ (s) Calculated	$T_{f}$ (s) Simulated	Filled?
0.2	26.7	179.4	1.4	1.422	Y
0.3	26.7	179.4	2.1	2.148	Y
0.35	26.7	179.4	2.45	3.205	N
	26.7	193.3	2.45	2.606	Y
0.375	26.7	193.3	2.625	3.479	N
	26.7	210	2.625	2.764	Y
	37.8	179.4	2.625	3.462	N
	37.8	193.3	2.625	2.963	Y
0.4	37.8	193.3	2.8	3.881	N
	37.8	210	2.8	3.132	Y
	48.9	179.4	2.8	3.735	N

## Data Availability

Data available from the authors.
